# MULTI-OMICS as Invaluable Tools for the Elucidation of Host–Microbe–Microbiota Interactions

**DOI:** 10.3390/ijms232113303

**Published:** 2022-11-01

**Authors:** Gary A. Toranzos, Tasha M. Santiago-Rodriguez

**Affiliations:** 1 Environmental Microbiology Laboratory, Biology Department, University of Puerto Rico, Rio Piedras Campus, San Juan 00931, Puerto Rico; 2 Diversigen Inc., Houston, TX 77046, USA

“Omics” is becoming an increasingly recognizable term, even to the general public, as it is used more and more often in everyday scientific research. It is also a means of understanding the biome and its interactions with the host and the environment [1,2,3]. This Special Issue primarily focuses on different aspects of the applications of “omics” in public health and microbial ecology, and it has offered a short preview of what is to come. The scientific and clinical community are becoming not only more adept in the use of “omics”, but also in implementing new methods of data acquisition and analysis, as well as cross-referencing data. As a result, “omics” is now almost as widespread as the polymerase chain reaction [3]. However, we are still a long way from incorporating these methods and analyses as a routine part of the clinical laboratory. Although “omics” are gaining acceptance in research compared to clinical ambience, we are still in the early stages of standardizing the integration of multiple “omics” (multi-“omics”) across studies, laboratories and institutions using ‘wet lab’ and bioinformatic tools [1].

The SARS-CoV-2 pandemic forced us to act quickly due to the demand for rapid results and answers in order to appropriately handle results demanded by the stakeholders, which in this case was everybody in the clinical, research, political and lay settings [4]. We were caught with our “omics” pants down, so as to speak. However, the industry eventually invented ingenuous equipment that required relatively little hands-on experience and expertise [5]. A few years ago, it would have been unbelievable to imagine the speed at which the first complete whole-genome sequences of some SARS-CoV-2 isolates would become available. All these data, in turn, were used as the framework for the development of mRNA vaccines, with results the whole world was able to see and be a part of [6,7]. One can already envision future approaches to many infections and diseases, especially the “forgotten” ones that affect the developing world.

The “omics” approach must be included in the study of the etiological agents of disease, as well as those agents that are beneficial to the biome [8]. Nothing in this world is void of biological entities, and these must be studied using “omics” approaches, if we are to completely understand what they do, how they do it, why they do it, and with whom they do it.

The present Special Issue is a good example of how we are learning to walk in this manner, and we are almost ready to run. Public health, clinical research (and somehow fundamental/basic research) and environmental health are intrinsically linked. The purpose of applying “omics” is an attempt to arrive at this scientific version of the Holy Grail.

For several decades, we have been aware of some microorganisms’ ability to be immunomodulatory to the host, but we never expected to find out the level of cross-talk between the microbiota and the host. One of the publications in this Special Issue reviews the current literature on how dysbiosis impacts chronic inflammatory diseases. La Barbera et al. discuss the role and interactions of the microbiota, short-chain fatty acid production and the metabolome in association with diseases such as systemic lupus erythematosus, Sjogren syndrome, systemic sclerosis, large vessel vasculitis and anti-neutrophil cytoplasmic antibody (ANCA)-associated vasculitis [9]. Ways to modulate the gut microbiota using probiotics, fecal transplants, and methotrexate in association with inflammatory diseases were also explored [9] (Figure 1).

Along similar lines, Purushothaman et al. review how whole-genome sequencing and metagenomics data can be combined for the purpose of microbial disease diagnosis to complement culture-based methods, which in turn pose their own limitations for diagnostics [10] (Table 1). Whole-genome sequencing and metagenomics are combined for microbiological diagnostics. The latter paper is key in terms of how to approach polymicrobial infections.

Indeed, multi-omics approaches are being applied in clinical settings to understand polymicrobial infections. This was the case for Silveira et al., who used a multi-“omics” approach in a case study of cystic fibrosis (CF) [11]. CF is complex, and patients suffer from chronic polymicrobial infections throughout their lives. In this study, Silveira et al. determined the state of a patient suffering from CF during and after antibiotic treatment to tease out how the microbiome was impacted by using a personalized multi-“omics” approach. Using quantitative metagenomics, transcriptomics and untargeted metabolomics, the authors aimed to determine how “typical” microorganisms, such as *Pseudomonas* spp., take advantage of the conditions for its growth and colonization, whereas antibiotic treatment lowered the numbers of some members of the microbiota, upon whose metabolic products *Pseudomonas* relied for its growth [11] (Figure 2). This study is intriguing, as it shows that personalized multi-“omics” may become a suitable approach for routine clinical diagnostics, providing critical information for treatment decision making.

Although virologists and bacteriologists remain convinced that pathogens can be attenuated through repeated passages in the laboratory, usually because of the loss of virulence factors, and some residing in mobile elements (as is the case in bacteria), little is known about this phenomenon in fungi. Breen et al.’s contribution focuses on a fungal phytopathogen (*Botrytis cinerae*), which is a pathogen of global concern [12]. Breen et al., give a plausible explanation (elucidated via whole genome (bisulfite) sequencing) that different levels in DNA methylation may be responsible for the virulence, or lack thereof in these pathogens, proposing a possible explanation for the existence of different pathovars within the same species, which can be reversible as a result of the levels of methylation [12] (Figure 3). Methylation is an extremely important part of the two-component regulatory system in bacteria (and plants); thus, data on this topic present a great opportunity for a different approach to perhaps fight phyto- (and maybe other) pathogens, as well as elucidating ways to modulate or control virulence.

Last, but not least, Jones et al. dare to investigate a topic that is usually confined to the realm of the quasidogmatic: herbal remedies to treat metabolic conditions, such as obesity and Type 2 diabetes [13]. Herbal remedies are considered by the lay public to be completely free of secondary effects and are usually self-administered ad libitum, which may be a risk if the modes of action are unknown. Jones et al. include data that indicate that the “real target” of some herbal remedies may be the microbiota, rather than the host. Specifically, the authors investigated how the use of fenugreek seeds altered the structure and function of the mice intestinal microbiome and metabolome. By using 16S rRNA high-throughput sequencing and untargeted metabolomics, the authors found metabolic processes affected by fenugreek. Several of the pathways impacted by fenugreek supplementation included carnitine biosynthesis, cholesterol and bile acid metabolism, and arginine biosynthesis. These results are intriguing, as they show that fenugreek may have beneficial effects in the treatment of metabolic conditions, something that also opens the door to the elucidation of many other questions on this topic.

Overall, this Special Section has been a great learning experience for all of us in several different areas of specific research, all linked by an incredible suite of “omics” methods. We hope that this selection of papers is as useful to the readers as it has been to us as researchers and graduate teachers.

## Figures and Tables

**Figure 1:**
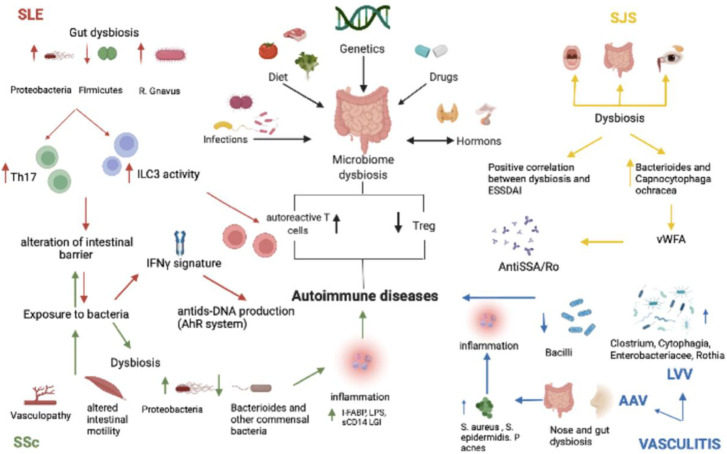
Main pathomechanisms in chronic inflammatory diseases as discussed by La Barbera et al. [9].

**Figure 2:**
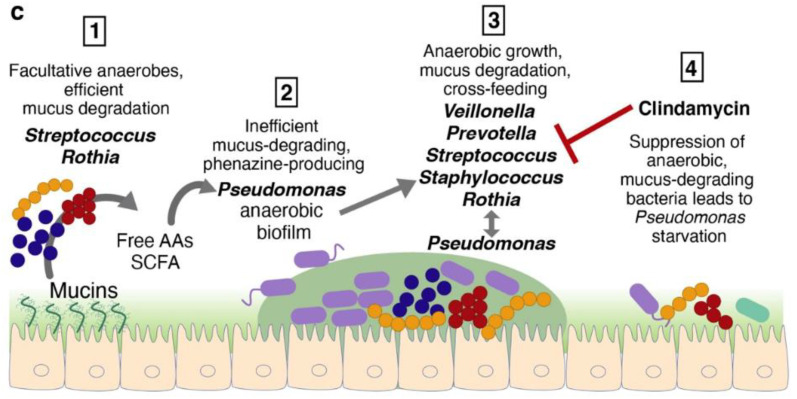
Conceptual model of succession events in a patient with cystic fibrosis from study. Modified from Silveira et al. [11].

**Figure 3:**
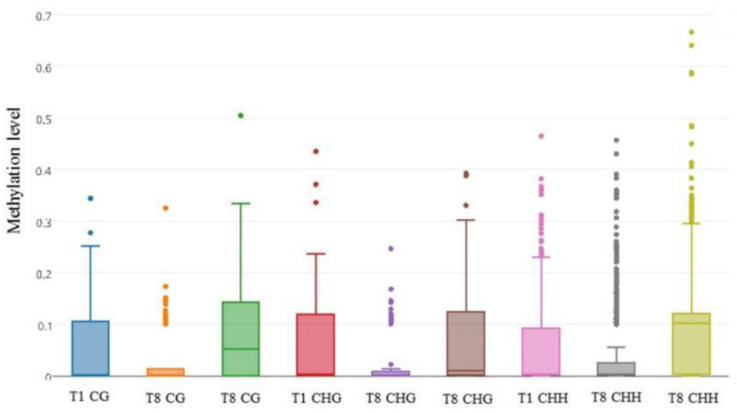
Methylation level distribution in in vitro-induced differentially methylated regions DMRs discussed Breen et al. [12].

**Table 1:** Comparison of whole-genome sequencing, marker gene-based amplicon sequencing (16S/ITS), and shotgun metagenomic sequencing, as discussed by Purushothaman et al. [10]. The symbol “$” represents sequencing costs, where a higher number of $ represents a higher cost. The symbol “+” represents the turnaround time for the sequencing strategies, where a higher number of + represents a longer turnaround time.

Parameters	WGS	16S/ITS	Shotgun Metagenomic Sequencing
Sample	Cultured or enriched microorganisms	Swabs from body sites, stool samples, body fluids or tissue samples, and sewage	Swabs from body sites, stool samples, body fluids or tissue samples fecal matter, and sewage
Species identification	Yes	Yes	Yes
Degree of resolution	Species-Strain level	Genus-Species level	Species-Strain level
Complete genome	Complete genome possible depending on sequencing platforms	No	Near complete to gapped genomes.
SNP analysis	Yes	No	Yes
GWAS	Yes	No	Yes
Identification of virulence factors and resistance genes	Yes	No	Yes
Microbial community profiling	No	Yes	Yes
Cost	$$	$	$$$
Turnaround Time (TAT)	+	++	+++
